# *Leishmania major* UDP-sugar pyrophosphorylase salvages galactose for glycoconjugate biosynthesis

**DOI:** 10.1016/j.ijpara.2015.06.004

**Published:** 2015-10

**Authors:** Sebastian Damerow, Carolin Hoppe, Giulia Bandini, Patricia Zarnovican, Falk F.R. Buettner, Michael A.J. Ferguson, Françoise H. Routier

**Affiliations:** a Hannover Medical School, Department of Cellular Chemistry OE4330, Carl-Neuberg-Strasse 1, 30625 Hannover, Germany; b Division of Biological Chemistry and Drug Discovery, College of Life Sciences, University of Dundee, Dundee DD1 5EH, Scotland, United Kingdom

**Keywords:** *Leishmania*, Trypanosomatid, Nucleotide-sugar, Galactose metabolism, Galactose salvage, Glycoconjugates

## Abstract

Leishmaniases are a set of tropical and sub-tropical diseases caused by protozoan parasites of the genus *Leishmania* whose severity ranges from self-healing cutaneous lesions to fatal visceral infections. *Leishmania* parasites synthesise a wide array of cell surface and secreted glycoconjugates that play important roles in infection. These glycoconjugates are particularly abundant in the promastigote form and known to be essential for establishment of infection in the insect midgut and effective transmission to the mammalian host. Since they are rich in galactose, their biosynthesis requires an ample supply of UDP-galactose. This nucleotide-sugar arises from epimerisation of UDP-glucose but also from an uncharacterised galactose salvage pathway. In this study, we evaluated the role of the newly characterised UDP-sugar pyrophosphorylase (USP) of *Leishmania major* in UDP-galactose biosynthesis. Upon deletion of the USP encoding gene, *L. major* lost the ability to synthesise UDP-galactose from galactose-1-phosphate but its ability to convert glucose-1-phosphate into UDP-glucose was fully maintained. Thus USP plays a role in UDP-galactose activation but does not significantly contribute to the de novo synthesis of UDP-glucose. Accordingly, USP was shown to be dispensable for growth and glycoconjugate biosynthesis under standard growth conditions. However, in a mutant seriously impaired in the de novo synthesis of UDP-galactose (due to deficiency of the UDP-glucose pyrophosphorylase) addition of extracellular galactose increased biosynthesis of the cell surface lipophosphoglycan. Thus under restrictive conditions, such as those encountered by *Leishmania* in its natural habitat, galactose salvage by USP may play a substantial role in biosynthesis of the UDP-galactose pool. We hypothesise that USP recycles galactose from the blood meal within the midgut of the insect for synthesis of the promastigote glycocalyx and thereby contributes to successful vector infection.

## Highlights

• Galactose salvage in *Leishmania major* is mediated by UDP-sugar pyrophosphorylase (USP).
• USP is not rate limiting for glycocalyx biosynthesis under standard growth conditions.
• Salvage by USP contributes to glycoconjugate biosynthesis but is insufficient on its own.

## Graphical abstract

**Figure:**
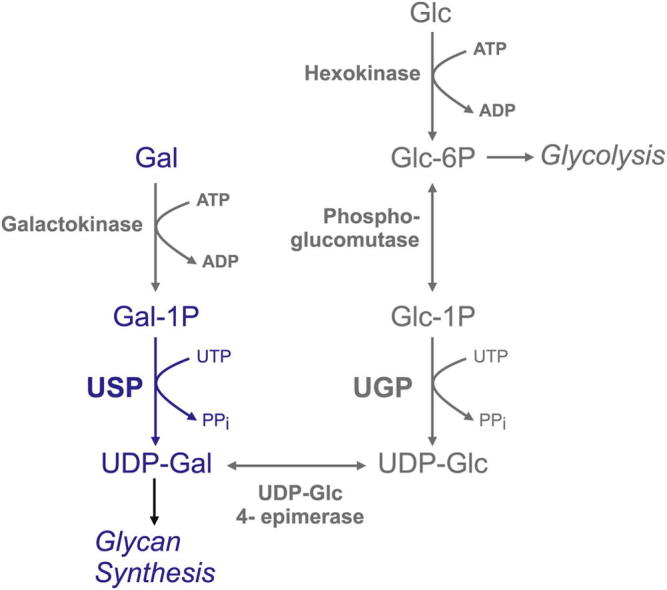

## 1 Introduction

Leishmaniases are a set of diseases caused by protozoan parasites of the genus *Leishmania* and transmitted by the bite of phlebotomine sandflies. The parasite presents itself in a motile elongated promastigote form that develops within the vector midgut and an immotile rounded amastigote form that multiplies within the phagolysosomes of host macrophages. The change in temperature and pH, encountered by amastigotes after ingestion by a female sandfly, triggers their differentiation into procyclic promastigotes that proliferate in the early blood meal. To successfully establish an infection of the posterior midgut, promastigotes need to resist proteolytic enzymes secreted for digestion of the blood meal and avoid excretion with the meal remnants, which requires their attachment to the midgut wall. Detachment, migration toward the anterior midgut and colonisation of the stomodeal valve are then needed for effective transmission to the vertebrate host. Infective metacyclic promastigotes that arise by a differentiation process called metacyclogenesis are delivered to the skin of the host during blood feeding. These differentiate into amastigotes that proliferate within macrophages and are responsible for disease progression in mammals (Sacks and Kamhawi, 2001, Dostalova and Volf, 2012).

Survival and development within the harsh milieus encountered by *Leishmania* are facilitated by a group of glycoconjugates present at the cell surface of the parasite or secreted in the environment. These are particularly abundant in promastigotes and accordingly glycans have been shown to play major roles within the insect and in the establishment of infection in vertebrates (Naderer et al., 2004). The roles of phosphoglycans (PGs) made of –6Gal(β1,4)Man(α1-PO_4_H)– repeat units have been particularly well studied. These repeat units form the abundant surface polysaccharide lipophosphoglycan (LPG), which is bound to the plasma membrane by a glycosylphosphatidylinositol (GPI)-anchor (McConville and Ferguson, 1993) or can be attached to the serine/threonine-rich regions of surface and secreted proteophosphoglycans (PPGs) (Ilg et al., 1996, Foth et al., 2002). Repeat units may be substituted by strain-, species- and stage-specific side chains β1,3-linked to the galactose residue (McConville et al., 1995). The structural changes of PGs associated with development of *Leishmania major*, and their importance for interaction with its specific vector *Phlebotomus papatasi*, have been well described. *Leishmania major* PGs predominantly exhibit galactose side chains that progressively become capped with β1,2-linked d-arabinose during parasite development within the insect gut (Saraiva et al., 1995, Dobson et al., 2010). The LPG galactose side chains have been shown to mediate attachment to a galectin present in the insect midgut epithelium (Kamhawi et al., 2004). Accordingly, an *L. major* mutant exclusively deficient in LPG failed to persist in its specific vector beyond the time of blood meal excretion (Sacks et al., 2000, Myskova et al., 2007). Capping with arabinose prevents recognition by galectin and thus enables parasite migration. PGs also play an important role in the protection of *Leishmania* against the hydrolytic enzymes of blood-fed phlebotomines. This protection was associated with surface acquisition of PG-containing molecules (Secundino et al., 2010). Finally, both LPG and PPG have also been shown to play a role in establishment of infection in the host (Spath et al., 2003, Rogers, 2012).

**Fig. 1:**
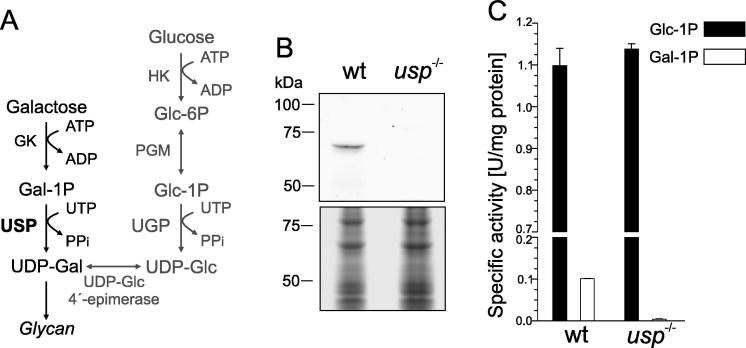
Absence of UDP-sugar pyrophosphorylase and UDP-galactose synthesis from galactose-1P-phosphate and uridine triphosphate in *Leishmania major usp*^−/−^ promastigotes. (A) De novo pathway (grey) for biosynthesis of UDP-galactose in *Leishmania* parasites and salvage pathway (black) inferred from this study. HK, hexokinase; PGM, phosphoglucomutase; GK, galactokinase. (B) Western blot of early log phase wild type (wt) and *usp*^−/−^ promastigote lysate probed with an anti-USP antiserum. Loading was assessed by Coomassie staining of an identically loaded SDS–PAGE ran separately. (C) In vitro conversion of glucose-1P (black bars) and galactose-1P-phosphate (white bars) into nucleotide sugar by cell lysates of wt and *usp*^−/−^ mutant. Standard deviation is indicated.

Biosynthesis of *Leishmania* glycoconjugates requires an ample supply of uridine diphosphate-α-d-galactose (UDP-Gal). In *L. major*, this nucleotide sugar is not only required for the synthesis of the PG repeat units but also for PG side-chains as well as glycoinositolphospholipids (GIPLs), whose role is less well defined (Zufferey et al., 2003, Kleczka et al., 2007). UDP-Gal is synthesized de novo by epimerization of UDP-α-d-glucose (UDP-Glc) by the UDP-glucose 4-epimerase (Fig. 1A). In the related parasite *Trypanosoma brucei* and *Trypanosoma cruzi*, this enzyme is the only route to UDP-Gal and is essential for parasite survival (Roper et al., 2005, MacRae et al., 2006, Urbaniak et al., 2006). In *L. major*, deletion of the specific UDP-Glc pyrophosphorylase (UGP) responsible for de novo synthesis of UDP-Glc and UDP-Gal revealed the presence of a redundant enzyme. However, in the UDP-Glc pyrophosphorylase null mutant the synthesis of these two nucleotide sugars was only sufficient to support GIPLs (and not LPG) synthesis (Lamerz et al., 2010).

Early radiolabelling experiments demonstrated the ability of *L. major* to import [^3^H]-galactose into cells and incorporate it into its glycoconjugates (Turco et al., 1984). This galactose salvage pathway distinguishes *Leishmania* from *T. brucei* and *T. cruzi*. The hexose transporters of *Leishmania* are able to import galactose in contrast to those of *T. brucei* and *T. cruzi* (Landfear, 2010). In most organisms, galactose salvage is enabled by the Leloir pathway. This pathway involves phosphorylation of α-d-galactose to α-d-galactose-1-phosphate (Gal-1P) by a galactokinase and production of α-d-glucose-1-phosphate (Glc-1P) and UDP-Gal from Gal-1P and UDP-Glc by the UDP-Glucose:α-d-galactose-1-phosphate uridylyltransferase. Glc-1P can subsequently be converted into Glc-6-phosphate (Glc-6P) by a phosphoglucomutase and enter glycolysis. Although a putative galactokinase gene can be identified in the *Leishmania* genome, no UDP-Glc:Gal-1P uridylyltransferase seems to be present, suggesting the absence of a Leloir pathway in this organism.

Recently, a UDP-sugar pyrophosphorylase (USP) homologous to plant USPs has been identified in *L. major* and *T. cruzi* and was shown to exhibit broad substrate specificity in vitro (Damerow et al., 2010, Yang and Bar-Peled, 2010). The *Leishmania* enzyme activated predominantly Gal-1P and Glc-1P in the presence of UTP to form the respective UDP-sugars but was unable to activate N-acetylglucosamine-1-phosphate (Damerow et al., 2010, Dickmanns et al., 2011). Here, we demonstrate that *L. major* USP is responsible for galactose salvage and contributes to glycoconjugate biosynthesis (Fig. 1A).

## 2 Materials and methods

### 2.1 Parasite culture and growth curves

Promastigote cultures of wild type *L. major* MHOM/SU/73/5ASKH and respective mutant cell lines were grown at 27 °C in standard culture medium consisting of M199 medium (Gibco®, Life Technologies, Darmstadt, Germany) supplemented with 10% heat inactivated foetal bovine serum (FBS) (Gibco®, Life Technologies, Darmstadt, Germany), 40 mM Hepes pH 7.5, 0.1 mM adenine, 0.0005% hemin, 0.0002% biotin. Parasite cultures were initiated frequently from frozen stocks. In order to test selective growth in different carbon sources, promastigotes were cultivated with sugar-deficient RPMI 1640 medium (Gibco®, Life Technologies, Darmstadt, Germany) supplemented with 10% dialyzed FBS and 50 mM of a carbohydrate source consisting of either glucose, galactose or a glucose/galactose mixture. Alternatively, sugar-deficient RPMI 1640 medium (Invitrogen) supplemented with 10% heat inactivated FBS and either 5 mM glucose, 5 mM galactose or 5 mM glucose and 5 mM galactose mixture was used. Antibiotics (InvivoGen, San Diego, USA) were used when appropriate at the following concentration: 5 μg/ml of phleomycin, 30 μg/ml of puromycin and 100 μg/ml of nourseothricin.

### 2.2 Genetic manipulation of *L. major*

The sequences of all primers used can be found in Supplementary Table S1. USP gene (LmjF*.*17.1160) replacement cassettes were constructed by double-joint PCR. First 2.3 kb of the 5′ untranslated region (UTR) and 1.3 kb of the 3′UTR were amplified with the primer pair 5UTR_1fw/5UTR_1rev and 3UTR_1fw/3UTR_1rev, respectively. The *ble* or *pac* gene conferring resistance to phleomycin or puromycin was amplified with primer pair OL_BLEfw/OL_BLErev or OL_PACfw/OL_PACrev having 52 bp homology to the 5′-UTR (forward primers) or 51 bp homology to the 3′-UTR (reverse primers). All PCR reactions (50 μL) were performed with 1 unit of Phusion® High-Fidelity DNA Polymerase (New England Biolabs, Ipswich, MA, USA), 200 μM dNTPs, 3% DMSO and 1 μM of each primer in Phusion high fidelity buffer. Amplification of the 5′ and 3′ untranslated regions and antibiotic resistance genes were performed using 150 ng of template DNA with the following reaction conditions: 98 °C for 2 min, followed by 30 cycles consisting of 96 °C for 10 s, 74 °C for 1 s, 61 °C for 30 s, 72 °C for 1 min, with a final extension at 72 °C for 5 min.

Fusion of the three amplicons was performed using PCR with a template DNA mix consisting of a molar ratio of 1:1:2 of the amplicons representing the 5′untranlated region, the 3′untranslated region and antibiotic resistance gene. PCR cycling conditions were 98 °C for 2 min followed by 15 cycles consisting of 98 °C for 10 s, 80 °C for 1 s, 65 °C for 10 min, 72 °C for 1 min 20 s, with a final extension at 72 °C for 10 min.

To generate each deletion cassette, the above PCR products were separately used as template in a nested PCR with the primer pair 5UTR_3fw/3UTR_3rev. The following PCR cycling conditions were used: 98 °C 2 min (Δ*T*/s = 4 °C/s) followed by 29 cycles consisting of 98 °C for 10 s (Δ*T*/s = 4 °C/s), 75 °C for 1 s (Δ*T*/s = 4 °C/s), 65 °C for 15 s (Δ*T*/s = 0.5 °C/s), 72 °C for 1 min 30 s T/s = 4 °C/s), with a final extension at 72 °C for 5 min (Δ*T*/s = 4 °C/s). Each deletion cassette was ligated to the vector, pYES-NTA, via *Not*I restriction sites to generate plasmids #3612 and #3613. All constructs were verified by sequencing. Before transfection into *L. major* promastigotes, the deletion cassettes were excised from each recombinant plasmid with *Bbv*CI and *Xcm*I, isolated via gel electrophoresis in 0.7% agarose in Tris–acetate EDTA (pH 8.0), ethanol precipitated and dissolved in water at a concentration of approximately 2 μg/μl. DNA transfection into *L. major* promastigotes was performed by electroporation, using the high voltage protocol and cytomix buffer as described previously (Robinson and Beverley, 2003). *usp*^−/−^ clones (Δ*usp*::*BLE*/Δ*usp*::*PAC*) were recovered after two consecutive rounds of homologous recombination on semi-solid plates containing 1% Noble agar and appropriate antibiotics.

Genomic DNA was isolated by phenol/chloroform extraction from log phase *L. major* promastigotes. Southern blots were performed using digoxigenin (DIG)-labelled probes, synthesized using the DIG DNA labelling mix (Roche, Mannheim, Germany), with primer pairs SD1/USP1_rev; SD176/SD21 and SD9/3UTR_4rev (Supplementary Table S1), according to the manufacturer’s instructions.

### 2.3 USP antibody preparation

Three New Zealand rabbits were immunised by s.c. injection with 500 μg of recombinant hexahistidine (His_6_)-tagged *L. major* USP purified as previously described (Damerow et al., 2010). For the first injection, His_6_-USP protein was mixed with FCA (Difco, Detroit, USA), followed by six injections at 6-week intervals using protein mixed with incomplete Freund’s adjuvant (Difco). Blood was collected 10 days after the last injection. Antiserum was diluted up to 1:20,000.

### 2.4 Western blotting

Early log phase promastigotes (approximately 1 × 10^8^ parasites) were lysed by sonication (Branson Sonifier 450, output cycle 50, 4 × 30 s) on ice in 100 μL of lysis buffer (50 mM Tris–HCl pH 7.8, 10 mM MgCl_2_, 1 mM phenylmethylsulfonyl fluoride (PMSF), 4 μM Leupeptin, 5 μM Pepstatin, 0.1% Triton X-100). Lysates were separated on SDS–PAGE and transferred onto polyvinylidene difluoride (PVDF) membranes. An equal protein load was analysed by staining with a mouse monoclonal anti-α-tubulin antibody (dilution 1:500) (Sigma) followed by anti-mouse IgG IR700 Dye 700 CW (dilution 1:20,000) (Li-Cor) or assessed by Coomassie brilliant blue protein staining of an identically loaded SDS–PAGE. Infrared detection on a Li-Cor Odyssey Imager was performed after incubation with monoclonal anti-LPG WIC79.3 antibody (protein G purified from mouse hybridoma cells) followed by incubation with goat anti-mouse IgG IR800 Dye 800 CW (Li-Cor) at dilutions of 1:1000 and 1:20,000, respectively. *Leishmania major* USP was detected using a 1:20,000 dilution of polyclonal rabbit anti-serum and goat anti-rabbit IgG IR800 Dye 800 CW (dilution 1:20,000) (Li-Cor).

### 2.5 In vitro enzyme assays

Log phase promastigotes (approximately 1 × 10^8^) were lysed by sonication on ice in 100 μL of lysis buffer (50 mM Tris–HCl pH 7.8, 10 mM MgCl_2_, 1 mM PMSF, 4 μM Leupeptin, 5 μM Pepstatin). Insoluble material was removed by centrifugation and the protein concentration in supernatant was determined by a Bradford Assay (BioRad, Munich, Germany). Enzymatic activity was tested in a 100 μL reaction volume in assay buffer (50 mM Tris–HCl, pH 7.8 with 10 mM MgCl_2_). Formation of UDP-Gal was measured in a preparation supplemented with 2 mM NAD+, 2 mM galactose-1-phosphate, 1 mM UTP, 0.12 units/ml of UDP-galactose 4-epimerase (GALE, *Streptococcus thermophilus*, Calbiochem, CA, USA) and 0.08 U/ml of UDP-glucose dehydrogenase (UGDH, bovine liver, Calbiochem). Similarly, UDP-Glc synthesis was measured using the assay buffer supplemented with 2 mM NAD^+^, 3 mM glucose-1-phosphate, 1 mM UTP and 0.08 U/ml of UGDH. Formation of NADH was detected at 340 nm. All measurements were performed in 96-well half-area flat-bottom microplates (Greiner Bio-One, Frickenhausen, Germany) with the Power-WaveTM340 KC4 System (Bio-Tek, Winooski, VT, USA). The activity was calculated according to Lambert–Beer’s law and units were normalised to the amount of protein (U/mg).

### 2.6 Quantification of the nucleotide sugar pools

Samples and data were processed as detailed in Turnock and Ferguson, 2007. In brief, 5 × 10^7^ exponentially growing promastigotes were harvested, washed with ice-cold PBS, lysed with 70% ethanol and spiked with 20 pmol of the internal standard GDP-glucose. The samples were lipid-extracted with butanol and the hydrophilic phase was used for nucleotide sugar extraction by Supelclean™ Envi™-Carb graphitized carbon columns (Supelco, Bellefonte, PA, USA). The eluted and freeze dried samples were separated by reverse-phase HPLC (Ultimate, Dionex, Idstein, Germany) on a 1 × 250 mm C18-column (HiChrom, Theale, UK) using a linear gradient of 0.5–4% acetonitrile in 20 mM triethylammonium acetate buffer (pH 6). Nucleotide sugars were detected by electrospray ionisation-tandem mass spectrometry (ESI-MS/MS) using multiple reactions monitoring (MRM) in negative ion mode on a Quattro Ultima triple quadrupole instrument (Waters, Milford, MA, USA). A standard master mix of nucleotide diphosphate sugars (NDP-sugars) with known absolute quantities was used as a reference on each day of analysis. Peak areas were integrated automatically by MassLynx software V4.1. The cellular pools of nucleotide sugars were calculated using the following formula: Quantity of NDP-sugar × = (sample area _NDP–sugar_/sample area _GDP–Glc_) * (standard area _GDP–Glc_/standard area _NDP–sugar_) * (20 pmol * coefficient _NDP–sugar_); with coefficient _NDP–sugar_ describing a multiple of GDP-Glc of the actual NDP-sugar concentration in the standard master mix.

### 2.7 GIPL analysis by MALDI-TOF-MS

A cell pellet of 4 × 10^8^ late log phase promastigotes was extracted in chloroform/methanol/water (5:10:4), purified over a C18/ SepPak® Plus column (Waters) and dried under a nitrogen stream as described previously (Kleczka et al., 2007). Cell equivalents (1.6 × 10^7^) of GIPLs extract dissolved in CHCl_3_/MeOH/H_2_O (15:30:4) were mixed equally with 6-Aza-2-thiothymidine matrix (5 μg/μl of H_2_O) and spotted on a metal target plate. MALDI-TOF-MS was carried out by using a Voyager DE Pro (Applied Biosystems, Foster City, CA, USA). Analyses of GIPLs were performed in negative-ion reflector mode over the *m*/*z* range 900–2000 with an accelerating voltage of 20 kV and a delay of 150 ns. The instrument was externally calibrated. A low-mass gate value of *m*/*z* 600 was selected to avoid saturation of the detector. Final mass spectra represented an average of 6–8 spectra, each of which is acquired from 200 laser shots. Spectra were processed using Data Explorer® Software V4.8 applying “Advanced Baseline Correction” and “Noise Removal”.

## 3 Results

### 3.1 *Leishmania major* USP is involved in synthesis of UDP-Gal from Gal-1P and UTP

**Supplementary Fig. S1:**
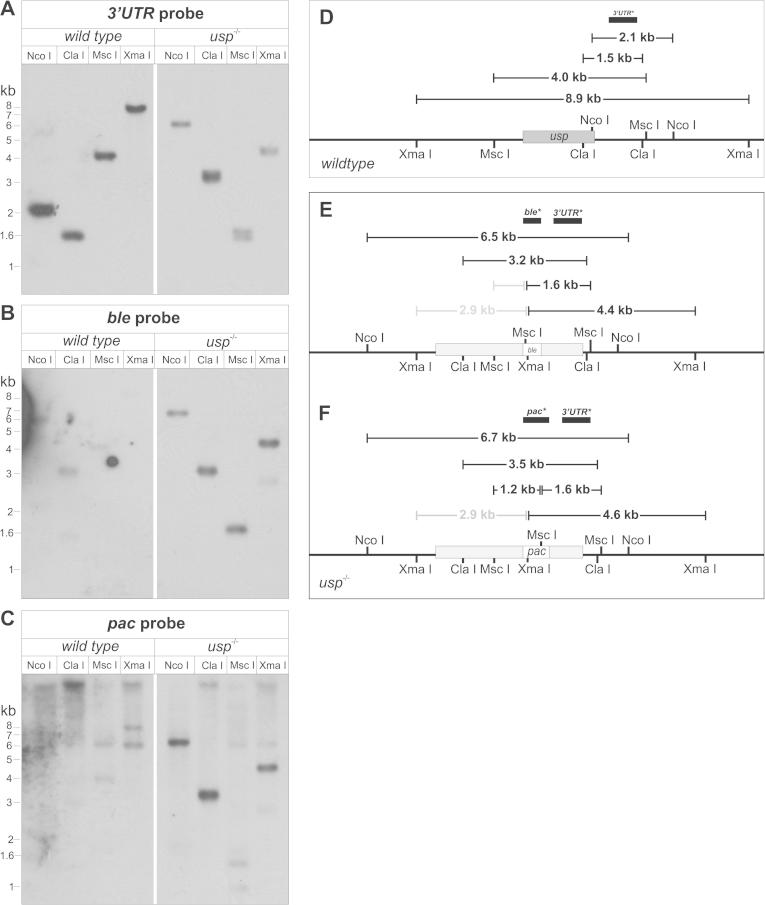
Southern blot analysis of *Leishmania major usp*^−/−^ mutant**. (**A–C) Genomic DNA from wild type (wt) and homozygous *usp*^−/−^ mutant (Δ*usp*::*BLE*/Δ*usp*::*PAC*) was digested with four different endonucleases, separated on agarose gel, blotted onto nylon membrane, and hybridized with digoxigenin labeled probes specific for *3*′*UTR^∗^*, *ble^∗^* and *pac^∗^*. The shifts of the probe labelled fragments can be assigned to the respective theoretical length in D–F, indicating correct gene replacement.

In order to assess the role of *L. major* USP, we aimed to delete the gene encoding this enzyme (LmjF.17.1160) by targeted replacement. The *usp* alleles were successively exchanged by genes conferring resistance to phleomycin and puromycin (Δ*usp*::*BLE*/Δ*usp*::*PAC*). Correct integration of the antibiotic resistance genes was verified by Southern blots (Supplementary Fig. S1) and the resulting mutant was named *usp*^−/−^.

To ascertain the absence of USP in this deletion mutant, lysates of wild type parasites and the *usp*^−/−^ mutant were first analysed by Western blot and probed with USP antiserum (Fig. 1B). As expected, a band at approximately 70 kDa confirmed expression of USP in wild type promastigotes whereas no USP was observed in the deletion mutant.

In vitro characterisation of *L. major* USP has demonstrated its ability to convert various sugar-1-phosphates into UDP-sugars (Damerow et al., 2010). Amongst the potential products of USP, only UDP-Glc and UDP-Gal (both in its pyranose and furanose form) were detected in *Leishmania* parasites (Turnock and Ferguson, 2007). To determine the contribution of USP to UDP-Gal and UDP-Glc biosynthesis, we analysed the ability of the *usp*^−/−^ mutant to activate Glc-1P and Gal-1P into the corresponding nucleotide sugars. Conversion of Glc-1P into UDP-Glc in promastigote lysate was not decreased in *usp*^−/−^ when compared with wild type parasites (Fig. 1C). This indicates that the contribution of USP to the de novo UDP-Glc biosynthesis is negligible. *Leishmania* parasites indeed possess a highly active and specific UDP-glucose pyrophosphorylase (UGP), ensuring this reaction (Lamerz et al., 2006, Lamerz et al., 2010). In contrast, synthesis of UDP-Gal from Gal-1P and UTP observed in the promastigote lysate is entirely due to USP since this activity is lost in the *usp*^−/−^ mutant (Fig. 1C).

### 3.2 *Leishmania major* USP is not essential for growth under standard culture conditions

**Fig. 2:**
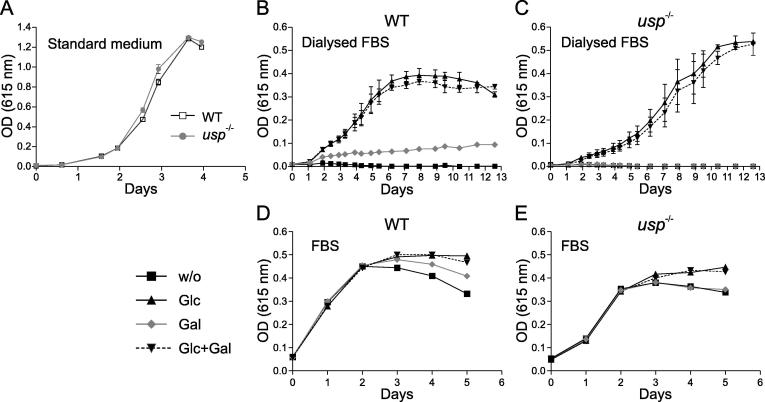
In vitro growth of *Leishmania major usp*^−/−^ mutant. (A) In vitro growth of wild type (wt) and *usp*^−/−^ promastigotes in standard media M199 plus 10% foetal bovine serum inoculated with 1 × 10^5^ cells. (B and C) In vitro growth of *L. major* wt (B) and *usp*^−/−^ (C) promastigotes in sugar-deprived RPMI medium supplemented with 5% dialyzed foetal bovine serum, and containing either no additional carbohydrate (w/o), glucose, galactose or both glucose and galactose (50 mM total final concentration of additional carbohydrate). Cultures were inoculated with 1 × 10^5^ cells. Standard deviation is indicated. (D and E) In vitro growth of *L. major* wt and *usp*^−/−^ promastigotes in sugar-deprived RPMI medium supplemented with 5% heat inactivated foetal bovine serum, and containing either no additional carbohydrate (w/o), glucose, galactose or both glucose and galactose (5 mM final concentration of additonal carbohydrate). Cultures were inoculated with 1 × 10^6^ cells.

The *usp*^−/−^ promastigotes were morphologically identical to the parental strain and grew at similar rates and density under standard culture conditions (Fig. 2A). As shown previously (Rodriguez-Contreras et al., 2007), wild type *Leishmania* grew to some extent in medium supplemented with dialysed FBS and galactose (50 mM) as the sole carbon source (Fig. 2B). In contrast, the *usp*^−/−^ mutant only showed residual growth in this medium (Fig. 2C), comparable to the growth observed when *usp*^−/−^ or wild type parasites are grown in medium without monosaccharides (Fig. 2B and C). In the latter case, the residual growth was previously attributed to gluconeogenesis (Rodriguez-Contreras et al., 2007). Similar results were obtained with parasites grown in sugar-deficient medium supplemented with heat inactivated FBS and hemin (Fig. 2D and E). As shown previously, nutrients present in the FBS (which includes ∼ 0.5 mM glucose) were depleted after approximately 3 days (Rodriguez-Contreras et al., 2015). However, glucose (5 mM) or to a lesser extend galactose (5 mM) were able to further support growth. As previously, growth of the *usp*^−/−^ clones was similar in medium supplemented with FBS only or with FBS and galactose (5 mM) (Fig. 2E) suggesting that the gain of energy from galactose (even if inadequate) is due to USP.

As shown above, USP is able to activate Gal-1P into UDP-Gal, which in turn can be epimerized in UDP-Glc. In contrast to animal cells and fungi, *Leishmania* store energy in the unusual form of mannogen (Ralton et al., 2003, Sernee et al., 2006) and is thus unable to obtain Glc-1P by breakdown of glycogen. However, phosphorolysis of UDP-Glc by UGP would provide a weak connection to the glycolytic pathway enabling restricted growth of the parasites when galactose is the only extracellular carbohydrate source. The inefficacy of such a pathway suggests, however, that gaining energy from galactose is not the primary function of USP.

**Supplementary Fig. S2:**
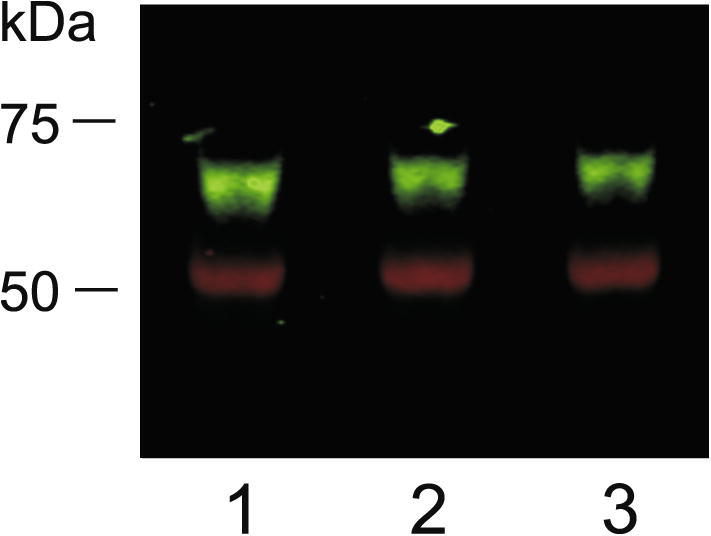
Level of UDP-sugar pyrophosphorylase (USP) in wild type *Leishmania major* grown with diverse carbohydrate sources. Lysates from early stationary phase *L. major* grown in sugar-deficient RPMI medium supplemented with 10% FBS and containing either no additional carbohydrate (lane 1), 10 mM galactose (lane 2) or 10 mM glucose (lane 3) were analysed by Western blot and labelled with anti-USP (green) or anti-α-tubulin (red) antibodies.

Since USP plays a role in galactose metabolism, we investigated whether the presence of galactose in the medium could influence the expression or stabilisation of USP. Therefore lysates of parasites grown in sugar-deficient RPMI medium (supplemented with 10% FBS) containing either no additional carbohydrate, 10 mM glucose or 10 mM galactose were analysed by Western blot. USP was strongly expressed in promastigotes regardless of the carbohydrate source (Supplementary Fig. S2).

### 3.3 USP is not rate limiting for glycocalyx biosynthesis under standard growth conditions

**Fig. 3:**
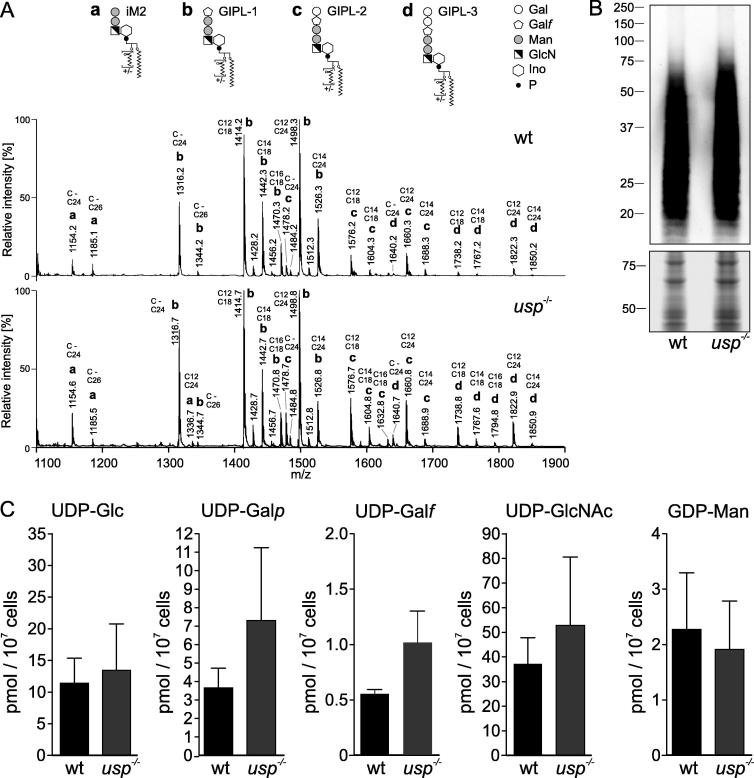
Analysis of cell surface glycosylation and nucleotide sugar pools in the *Leishmania major usp*^−/−^ mutant grown under standard conditions. (A) Negative-ion MALDI-TOF-MS of glycosylinositolphospholipids isolated from wild type (wt) and *usp*^−/−^ mutant. Each prominent peak is annotated with its *m*/*z* value, the letter a, b, c or d (lowercase in bold) referring to the glycosylinositolphospholipid structure depicted above (iM2, GIPL-1, GIPL-2 GIPL-3), and the length of acyl and alkyl chains. Gal, galactose; Gal*f*, galactofuranose; Man, mannose, GlcN, galactosamine; Ino, inositol; P, phosphate; (B) Western blot of log phase wt and *usp*^−/−^ mutant probed with the anti-lipophosphoglycan monoclonal antibody WIC79.3. Loading was assessed with Coomassie staining of an identically loaded SDS–PAGE run separately. (C) Nucleotide sugar pools of mid-log phase wt and *usp*^−/−^ promastigotes measured in cell extracts by liquid chromatography-electrospray ionisation-tandem mass spectrometry with multiple reaction monitoring. Standard deviation is indicated. UDP-Gal*p*, UDP-galactopyranose; UDP-Gal*f*, UDP-galactofuranose; UDP-GlcNAc, UDP-N-acetylglucosamine; GDP-Man, GDP-mannose.

In order to test whether USP can affect the glycocalyx formation, GIPLs and LPG from wild type *L. major* and the *usp*^−/−^ mutant were analysed. GIPLs were purified from late-log phase promastigotes cultured in standard medium and subjected to negative-ion MALDI-TOF-MS. The obtained mass spectra (Fig. 3A) were annotated according to structures previously described (McConville and Ferguson, 1993). *Leishmania major* produces a dense coat of type-2 GIPLs containing the core structure Gal*ƒ*(β1–3)Man(α1–3) Man(α1–4)GlcN(α1–6)phosphatidylinositol (PI). If it is not further elongated, this core structure is termed GIPL-1. It can however be substituted by one or two galactopyranose residues linked to the terminal galactofuranose (Gal*ƒ*) residue to generate GIPL-2 and GIPL-3, respectively (GIPL-2: Gal(α1–3)Gal*ƒ*(β1–3)Man(α1–3)Man(α1–4)GlcN(α1–6)PI; GIPL-3: Gal(α1–6)Gal(α1–3)PI). The carbohydrate moieties are linked via an inositolphosphate to an alkylacyl- or lysoalkylglycerol with saturated fatty acids of various lengths, which give rise to further heterogeneity. Importantly no major difference in the peak pattern obtained from *usp*^−/−^ and wild type GIPLs were observed (Fig. 3A).

Furthermore, analysis of LPG in wild type and *usp*^−/−^ promastigote lysate by Western blot stained with the monoclonal antibody WIC79.3 (recognising the galactose side chains decorating the LPG backbone) did not reveal any decrease in the abundance or size of LPG (Fig. 3B). This result was anticipated since the enzymatic conversion of Glc-1P into UDP-Glc was not decreased in the *usp*^−/−^ (Fig. 1C) suggesting that in standard medium, UGP alone is sufficient to fuel the nucleotide sugar pools and ensure glycocalyx biosynthesis.

In agreement with these results, analysis of the nucleotide sugar pools by liquid chromatography-ESI-MS/MS in wild type and *usp*^−/−^ promastigotes did not show any significant changes in the pools of UDP-Glc and UDP-Gal (either in the pyranic or furanic form) (Fig. 3C).

### 3.4 *Leishmania major* USP salvages extracellular galactose for glycoconjugate biosynthesis

**Fig. 4:**
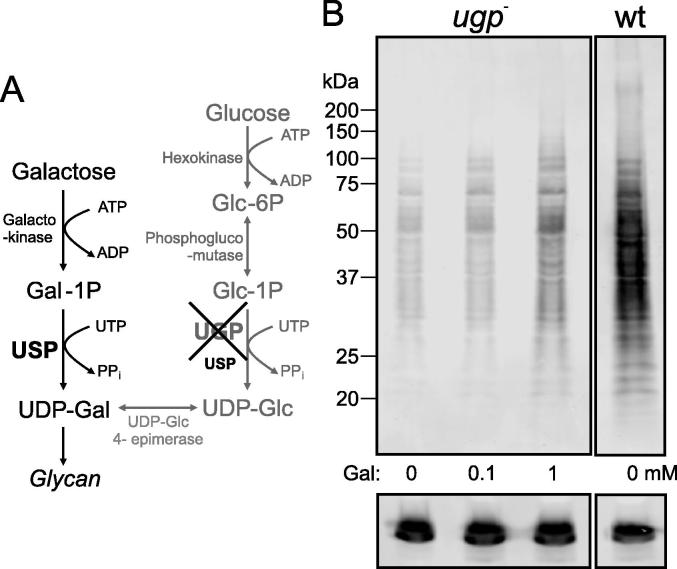
Analysis of lipophosphoglycan in the UDP-glucose pyrophosphorylase deficient *Leishmania major ugp*^−^ mutant grown in medium containing extracellular galactose. (A) Scheme of the de novo and salvage pathway for UDP-galactose synthesis in the *ugp*^−^ mutant. Extracellular galactose imported via the hexose transporters may be activated to UDP-galactose via UDP-sugar pyrophosphorylase. (B) Western blot of lysates obtained from log phase *ugp*^–^ mutant grown in standard medium without galactose (lane 1) or with 0.1 mM (lane 2) or 1 mM galactose (lane 3) and from log phase wild type parasites (wt) grown in standard medium without galactose (lane 4). Lanes originate from the same Western blot probed with the anti-lipophosphoglycan monoclonal antibody WIC79.3. Loading was assessed with an anti-tubulin antibody. Gal-1P, α-d-galactose-1-phosphate; Glc-6P, α-d-glucose-6-phosphate.

In order to understand the role of USP in glycoconjugate biosynthesis, we used a mutant severely impaired in UDP-Glc/UDP-Gal de novo biosynthesis due to deletion of UGP (Fig. 4A). When this *ugp*^−^ mutant was grown in standard media, the GIPLs were unaffected but only approximately 15% of LPG was synthesised, indicating a reduction of the UDP-Glc/UDP-Gal supply (Lamerz et al., 2010). Here the *ugp*^−^ mutant was cultivated in medium supplied with 100 μM or 1 mM galactose and LPG was analysed by Western blot using WIC79.3 for detection. As shown in Fig. 4, LPG was increased when 100 μM extracellular galactose was provided and further increased with the addition of 1 mM galactose to the medium. However the wild type LPG level could not be reached, indicating that USP-dependent salvage alone is insufficient for LPG biosynthesis.

## 4 Discussion

Early experiments have demonstrated the ability of *Leishmania* parasites to take up [^3^H]-galactose and incorporate it into glycoconjugates, highlighting the existence of a galactose salvage pathway (Turco et al., 1984). The present study establishes that USP is the only enzyme able to activate Gal-1P into UDP-Gal in *L. major* and is thus responsible for galactose salvage. In line with this role, USP is dispensable for in vitro growth, although it may support a restricted growth if galactose is the only monosaccharide source.

In a rich environment, USP may contribute but is not essential for synthesis of the UDP-Glc and UDP-Gal pool necessary for glycoconjugate biosynthesis. UDP-Gal arises from de novo synthesis of UDP-Glc by the specific and highly active UGP and subsequent epimerization of UDP-Glc into UDP-Gal. As a consequence, deletion of USP had no influence on the glycocalyx formation when parasites were cultivated in standard medium (containing glucose). This result is in perfect agreement with our previous study, which showed that parasites deficient in UGP only retained a limited ability to convert Glc-1P into UDP-Glc, designating UGP as the central enzyme for de novo UDP-Glc/UDP-Gal biosynthesis (Lamerz et al., 2010).

The salvage of monosaccharides imported from the extracellular milieu or derived from the degradation of lysosomal glycoconjugates is used for the biosynthesis of glycans by a variety of organisms from bacteria to human (Tettamanti et al., 2003, Coyne et al., 2005, Freeze and Elbein, 2009). Here, using a mutant impaired in the de novo synthesis of UDP-Glc/UDP-Gal (Lamerz et al., 2010), we demonstrate that USP may use extracellular galactose for synthesis of glycoconjugates. This salvage pathway could contribute to the biosynthesis of glycoconjugates in the natural habitat of *Leishmania* and may be particularly important when glucose is limiting.

Amastigotes that are proliferating in mammalian phagocytic host cells reside within a sugar-poor parasitophorous vacuole and have a minimalist surface glycocalyx (McConville and Naderer, 2011). USP may thus be dispensable for this parasitic stage. In contrast, promastigotes evolving within the insect gut synthesise large quantities of galactose-rich glycoconjugates (Naderer et al., 2004). Since many glycosidases (including β-galactosidase and α-mannosidase) are present in the midgut of the sandfly, *P. papatasi* (Jacobson et al., 2007), galactose could be released from glycoconjugates present in the blood. Salvage of galactose would lower the requirement for de novo synthesis of UDP-Gal, thereby increasing Glc-6P availability for glycolysis. Synthesis of the promastigote glycocalyx and especially of phosphoglycans is critical for successful colonisation of the sandfly midgut. The phosphoglycans and particularly PPGs have also been shown to protect procyclic promastigotes from the digestive enzymes released in the midgut lumen (Secundino et al., 2010). Furthermore, LPG is a virulence factor involved in the establishment of infection in a mammalian host (Spath et al., 2003). Consequently, a substantial reduction in the UDP-Gal biosynthesis leads to a reduction in virulence (Lamerz et al., 2010).

The role of USP in diverse organisms likely depends on the availability of monosaccharides and the presence of specific sugar kinases for their activation. In plants, USP enables recycling of specific monosaccharides originating from degradation of cell wall polymers during growth. *Arabidopsis thaliana* USP has been shown to play a central role in the biosynthesis of UDP-L-arabinose for synthesis of the plant cell wall (Geserick and Tenhaken, 2013). Here, we demonstrate that salvage of galactose by USP contributes to the cellular pool of UDP-Gal and glycoconjugates biosynthesis in *L. major*. In other parasites, the in vivo substrates of USP remain to be defined.

## Appendix A Supplementary data

Supplementary Table S1 Primer sequences used in this study.
