# Supplementary material for: Leishmania major UDP-sugar pyrophosphorylase salvages galactose for glycoconjugate biosynthesis
Source: Int J Parasitol. 2015 Oct;45(12):783–90. doi: 10.1016/j.ijpara.2015.06.004 (PMC4722067; doi:10.1016/j.ijpara.2015.06.004)
Supplement: Supplementary Table S1 — Primer sequences used in this study. [file mmc1.docx]

**Supplementary Table S1.** Primer sequences used in this study.

| Primer name | Sequence (5’3’) 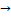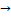 |
| --- | --- |
| OL-PAC fw | TTCCCCCCGCCGAGCCCCTCTGCTCTCTCCTTTTCTGTCGTCACGCGGCCTTATGACCGAGTACAAGCCCACGG |
| OL-PAC rev | CATTCAACTACACTGGAACACCCACACTAGCAAGGGCCCTCTCAACAACAATCAGGCACCGGGCTTGCGGGTC |
| OL-BLE fw | TTCCCCCCGCCGAGCCCCTCTGCTCTCTCCTTTTCTGTCGTCACGCGGCCTTATGGCCAAGTTGACCAGTGC |
| OL-BLE rev | CATTCAACTACACTGGAACACCCACACTAGCAAGGGCCCTCTCAACAACAATCAGTCCTGCTCCTCGGCCACG |
| 5UTR_1 fw | CTGACTGAGCGGCCGCTTGCTGATGAGGGAAGGATCTGC |
| 5UTR_1 rev | AAGGCCGCGTGACGACAGAAAAGG |
| 3UTR_1 fw | TTGTTGTTGAGAGGGCCCTTGC |
| 3UTR_1 rev | CTGACTGAGCGGCCGCACAGGAGCGACCTGCGACGACG |
| 5UTR_3 fw | CTGACTGAGCGGCCGCACGGTGCTGAGGACTGCG |
| 3UTR_3 rev | CTGACTGAGCGGCCGCTGCTGCAGCTCTGGCGAGC |
| SD1 | ATGACGAACCCGTCCAACTC |
| USP1rev | CCGGAGGTAGTAGGCGAGATA |
| SD176 | ACTGCATATGATGTTTTCGTGCAGCTCC |
| SD21 | AGGCCGCGTGACGACAGAAAAGG |
| SD9 | TGACGGGTGTCGGCCACACG |
| 3UTR_4rev | TGTCTGGGGCGCAGGCCGC |

Restriction sites are underlined.
